# Loss of SVIP Results in Metabolic Reprograming and Increased Retention of Very-Low-Density Lipoproteins in Hepatocytes

**DOI:** 10.3390/ijms26157465

**Published:** 2025-08-01

**Authors:** Vandana Sekhar, Thomas Andl, Shadab A. Siddiqi

**Affiliations:** 1 Division of Metabolic and Cardiovascular Sciences, Burnett School of Biomedical Sciences, College of Medicine, University of Central Florida, Orlando, FL 32827, USA; vandana.sekhar@ucf.edu; 2 Burnett School of Biomedical Sciences, University of Central Florida, 12722 Research Pkwy, Orlando, FL 32826, USA; thomas.andl@ucf.edu

**Keywords:** VLDL secretion, SVIP, PPARs, lipids, triglyceride, dyslipidemia

## Abstract

Perturbations in the tightly regulated processes of VLDL biosynthesis and secretion can directly impact both liver and cardiovascular health. Patients with metabolic disorders have an increased risk of developing hepatic steatosis, which can lead to cirrhosis. These associated metabolic risks underscore the importance of discerning the role of different cellular proteins involved in VLDL biogenesis, transport, and secretion. Small VCP-Interacting Protein (SVIP) has been identified as a component of VLDL transport vesicles and VLDL secretion. This study evaluates the cellular effects stemming from the CRISPR-Cas9-mediated depletion of SVIP in rat hepatocytes. The SVIP-knockout (KO) cells display an increased VLDL retention with elevated intracellular levels of ApoB100 and neutral lipid staining. RNA sequencing studies reveal an impaired PPARα and Nrf2 signaling in the SVIP KO cells, implying a state of metabolic reprograming, with a shift from fatty acid uptake, synthesis, and oxidation to cells favoring the activation of glucose by impaired glycogen storage and increased glucose release. Additionally, SVIP KO cells exhibit a transcriptional profile indicative of acute phase response (APR) in hepatocytes. Many inflammatory markers and genes associated with APR are upregulated in the SVIP KO hepatocytes. In accordance with an APR-like response, the cells also demonstrate an increase in mRNA expression of genes associated with protein synthesis. Together, our data demonstrate that SVIP is critical in maintaining hepatic lipid homeostasis and metabolic balance by regulating key pathways such as PPARα, Nrf2, and APR.

## 1. Introduction

The synthesis, intracellular transport, and secretion of very-low-density lipoproteins (VLDLs) from hepatocytes is a highly complex and tightly regulated process, crucial for maintaining overall lipid homeostasis. Increased biogenesis of VLDLs resulting in enhanced secretion of low-density lipoproteins (LDLs) into the circulatory system is one of the major contributing factors for the development of atherosclerosis [1]. On the contrary, an imbalance between the production of VLDLs and their secretion from the liver could lead to adverse outcomes such as hepatic steatosis [2]. Within hepatocytes, the endoplasmic reticulum (ER) is a site for hepatic lipid metabolism, including VLDL biogenesis [3]. Newly synthesized VLDL particles in the ER lumen are packaged into specialized large ER-derived vesicles, called VLDL transport vesicles (VTVs), which are subsequently delivered to the Golgi [4]. Akin to other intracellular ER-derived secretory vesicles like the protein transport vesicle (PTV) and pre-chylomicrons transport vesicle (PCTV), VTVs are characterized by the presence of coat complex proteins II (COPII) consisting of five cytosolic proteins, namely Sar1, Sec23-Sec24, and Sec13-Sec31 [5,6,7]. However, a thorough proteomic analysis of the VTVs from our laboratory has identified many additional proteins, such as reticulon 3, cell-death-inducing DFF45-like effector b (CideB). and Small VCP-Interacting Protein (SVIP), which are uniquely associated with the VTVs, likely contributing to their larger size and cargo selectivity [8].

Proteins like CideB are involved in secretion of VLDLs in addition to being involved in VTV biogenesis via its interaction with VLDL cargo protein apoB100 and COPII protein Sar1 [9]. In fact, CideB-/- mice have been reported to exhibit reduced VLDL secretion [10]. Similarly, biochemical and morphological studies have established SVIP as another integral component of VTVs [11]. Specifically, our laboratory has demonstrated that SVIP is enriched in VTVs and specifically interacts with ApoB100 and the COPII component Sar1b, like CideB. Interestingly, increased myristoylation of SVIP following incubation of primary hepatocytes with myristic acid resulted in enhanced VTV budding from the ER due to enhanced recruitment of SVIP and Sar1 to the ER membrane. Furthermore, the knockdown of SVIP using siRNA in hepatocytes demonstrated a significant reduction in VLDL secretion [11]. These findings support the idea that intracellular transport of VTVs from the ER to Golgi is a crucial and rate-limiting step in maintaining an overall balanced rate of VLDL secretion; hence, the presence of multiple proteins with redundant functions in this process ensures their compensatory role in the absence of any single protein.

In the current study, we generated a CRISPR-Cas9-mediated SVIP-knockout rat hepatoma cell line with the goal of further understanding the functional role of SVIP in the complex process of VLDL transport and secretion. We observed that complete absence of SVIP protein resulted in enhanced retention of VLDLs in the hepatoma cells. This was marked by both an increased expression of ApoB100 protein and neutral lipid staining detected inside SVIP-knockout (SVIP KO) cells. Furthermore, RNA sequencing studies of the SVIP KO hepatoma cells revealed a significant downregulation of genes within the peroxisome proliferator-activated receptor alpha (PPARα) and the nuclear factor erythroid 2-related factor 2 (Nrf2) pathways, thereby affecting hepatic fatty acid (FA) metabolism. Most importantly, we observed that cells lacking SVIP demonstrated a substantial downregulation of another important gene associated with FA metabolism, namely, the liver fatty acid-binding protein (L-FABP). Additionally, the RNAseq data also reveals that the loss of SVIP thrusts the hepatoma cells into an acute phase response (APR)-like state, with an increase in the expression of protein synthesis genes. Thus, the evidence reported in this study deems SVIP to be an integral part of the cellular lipid homeostasis machinery that also additionally regulates crucial cellular pathways, namely, PPARα, Nrf2, and APR.

## 2. Results

### 2.1. Generation of CRISPR-Cas9-Mediated SVIP-Knockout Rat Hepatoma Cells

The rat SVIP, a small 9 KDa protein, was discovered using a yeast two-hybrid system as a novel interacting adaptor for the multifunctional VCV/p97 protein [12]. The myristoylation site at the N terminus of SVIP contributes to its ER membrane affinity. Overexpression of SVIP has been associated with the formation of large aberrant vacuoles derived from the ER membrane [12]. SVIP has been demonstrated to be multifunctional in nature, from being an endogenous inhibitor of endoplasmic-reticulum-associated protein degradation (ERAD) to being a regulator of autophagy, in addition to being identified as a cellular factor for VLDL secretion [11,13].

**Figure 1:**
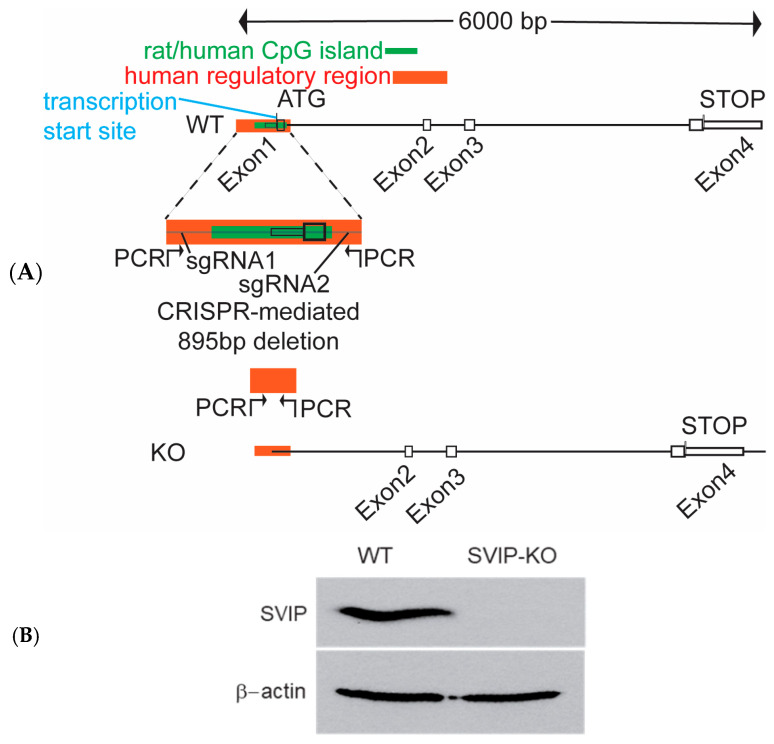

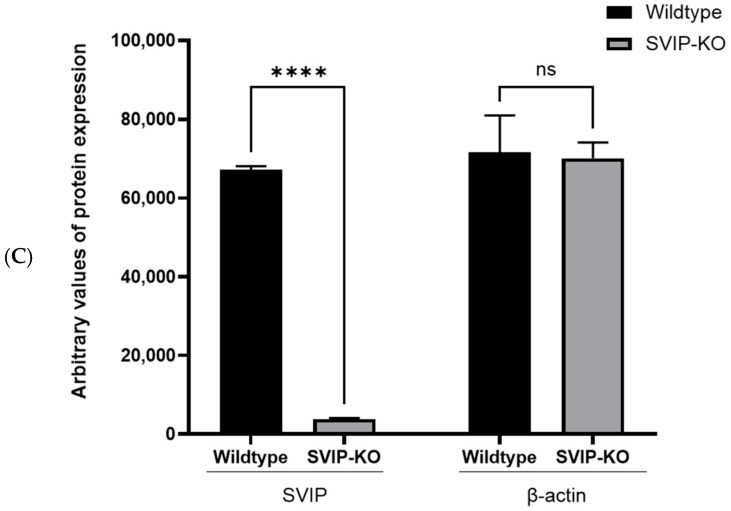
CRISPR-Cas9-mediated mutagenesis results in generation of SVIP-knockout rat hepatoma cell line. (**A**) Schematic diagram showing the region of the rat SVIP gene that was targeted for the CRISPR mutagenesis study. Two guide RNAs (sgRNA1 and sgRNA2) targeting the region around exon 1, including the promoter region, the CpG island, and parts of intron 1 in the wildtype cells, are shown. The CRISPR-Cas9-mediated mutagenesis resulted in a deletion of 895 bp with the majority of exon 1 deleted. (**B**) Western blot image confirming the complete loss of the SVIP protein in the SVIP KO cells compared to wildtype (WT) cells. β-actin was used as a loading control. (**C**) Relative expression levels of the SVIP and the β-actin proteins in the wildtype and SVIP KO cells. The data are representative of mean ± SD of three independent experiments. (**** *p* <0.0001, using unpaired *t*-test, ns= not significant).

To investigate the functionality of the SVIP gene in VLDL transport in depth, we performed a CRISPR-Cas9 mutagenesis. For designing a knockout (KO) strategy in a rat hepatoma model, we used the rat SVIP gene according to the Genome Browser for the Rat genome from July 2014 (Rat RGSC 6.0/rn6; chromosome 1:107,368,134-107,374,302). The exon/intron structure is based on the cDNA RefSeq NM_001271084.1 Based on https://www.fruitfly.org/seq_tools/promoter.html (accessed on 25 February 2022), the core promoter region spans from 215–165 bp upstream of the ATG start codon. A CpG island engulfs the entire Exon 1 region (ca. from 100 bp downstream and 330 bp upstream of the ATG codon). Due to the lack of data on the regulatory sequences in the rat SVIP gene, we used the human genome data, which shows a candidate cis-regulatory region spanning from ca. 400 bp upstream and 1200 bp downstream of the human ATG start codon. Based on these data on regulatory regions controlling the SVIP mRNA expression, two sgRNAs were designed to result in a ca. 888 bp deletion (in the actual clone, the deletion is 895 bp) around exon 1, including the promoter region, the CpG island, and parts of intron 1. The first sgRNA (sgRNA1-CCGCTCCCCTCGCCATTTAG) starts 616 bp upstream of the ATG codon, while the second sgRNA (sgRNA2-ATCAGTGGGGCAACGATGCG) is in intron 1 starting 292 bp downstream from the ATG (Figure 1A). Consequently, the deletion resulting from using both the sgRNAs simultaneously spans the entirety of exon 1, the core promoter region, and removes over 50% of the regulatory sequences identified in the human SVIP gene based on human ENCODE, including the most pronounced DNAse1-hypersensitive sites and most of the H3K4m1, H3K4m3, H3K27acylated regions. Thus, the described sgRNA designs delete most of the key regulatory sequences required to produce SVIP mRNA. Precisely, using PCR primers, the deletion was confirmed by comparing the size of the PCR product obtained from wildtype cells (1147 bp) to that of the mutant KO clone (252 bp).

In addition, as observed in Figure 1B,C, Western blot analysis further established the KO clone to be a complete SVIP-knockout cell line, as no SVIP protein was detected in these cells.

### 2.2. CRISPR-Cas9-Mediated SVIP Knockout Results in Increased VLDL Retention in Hepatoma Cells

**Figure 2:**
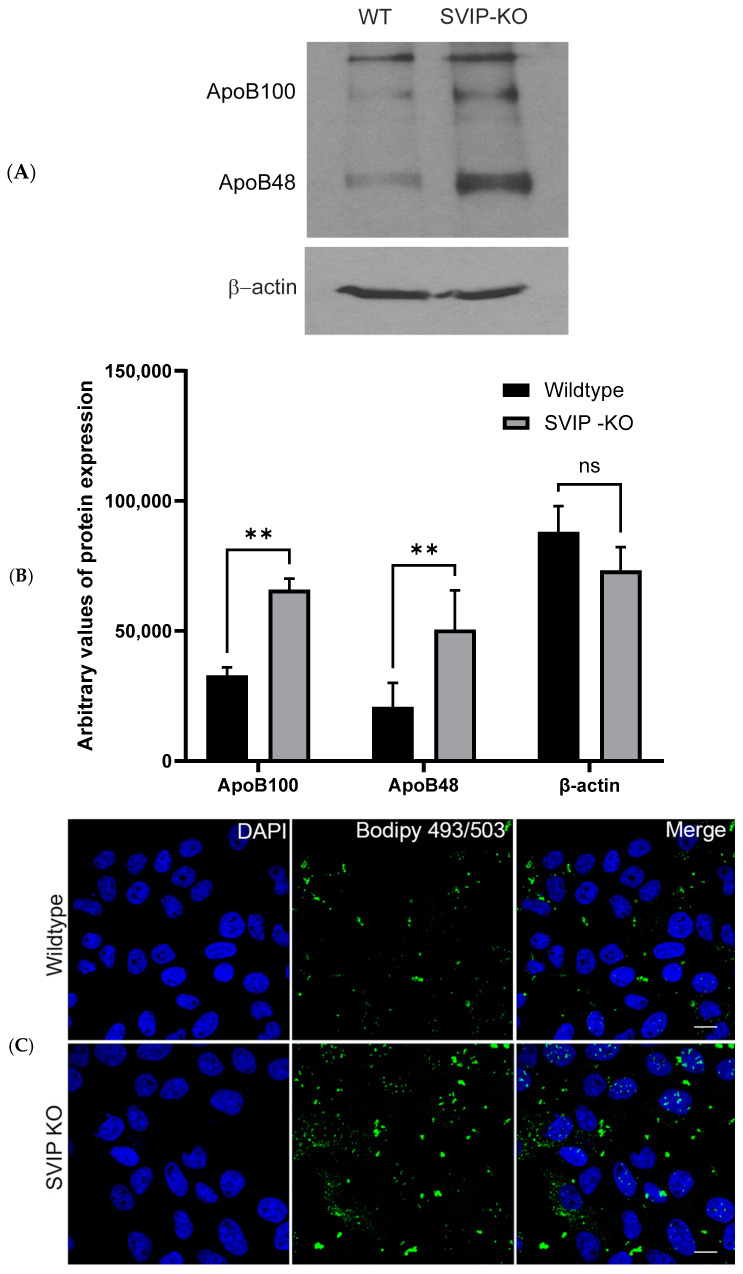

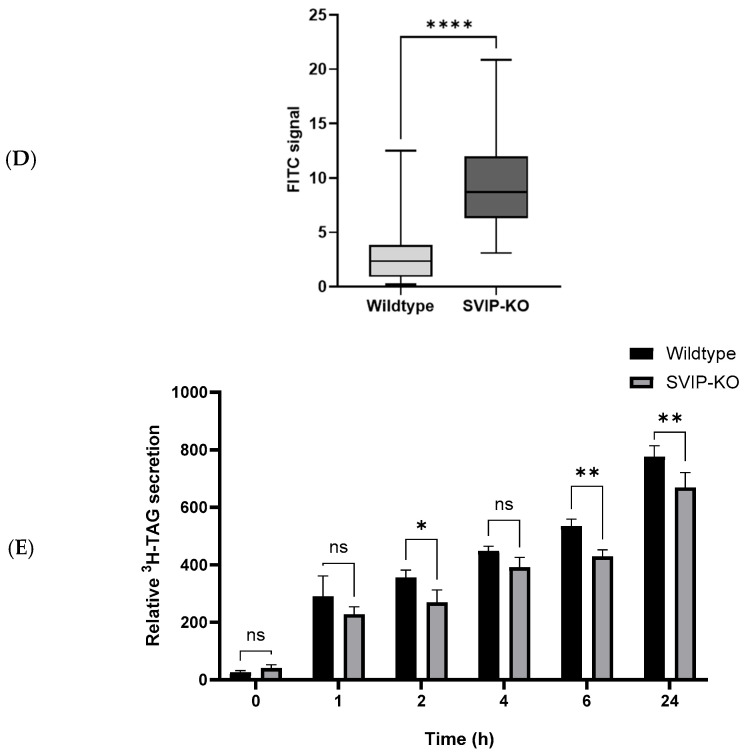
The SVIP KO cells exhibit an increased retention of VLDL compared to wildtype cells. (**A**) Increased expression of the ApoB100 and the ApoB48 proteins was detected in the SVIP KO cells using Western blot analysis. β-actin was used as a loading control. (**B**) Relative levels of expression of the ApoB100, ApoB48, and β-actin proteins in the wildtype and SVIP KO cells. The data are representative of mean ± SD of three independent experiments. (** *p* = 0.0028 for ApoB100, ** *p* = 0.0062 for ApoB48, and ns = not significant, using 2-way ANOVA). (**C**) Confocal images showing intracellular neutral lipid staining using the BODIPY 493/503 stain in the wildtype and the SVIP KO cells. The BODIPY 493/503 staining is shown in green, and the DAPI-stained nuclei are shown in blue. Images were taken using a 63x objective. Scale bar = 10 µm. (**D**) Quantification of the number of green spots (FITC signal) observed in wildtype and SVIP KO cells (**** *p*  <  0.0001, using unpaired *t*-test). (**E**) Quantification data showing relative levels of VLDL secretion from cells. Wildtype and SVIP KO cells were washed with warm (37 °C) PBS and incubated with [3H]-oleic acid complexed with BSA at a final concentration of 0.4 mM oleic acid for 1 h. Post incubation, medium containing oleic acid–BSA was removed and replaced with fresh medium without oleic acid–BSA complex. A total of 200 µL of medium was collected at different time points, and the resulting associated [^3^H]-TAG dpm was measured using scintillation counter. Error bars represent mean ± S.D. (*n* = 3), (* *p* = 0.0411, ** *p* = 0.0085 for 6 h, ** *p* = 0.0070 for 24 h, and ns = not significant, using 2-way ANOVA).

A study from our laboratory has established the important role of SVIP in intracellular VLDL trafficking and secretion [11]. A radioactivity assay measuring [^3^H]-triacylglycerol (TAG) secretion and ApoB100 protein levels in the medium is employed to monitor VLDL secretion. The ApoB100 protein is a critical protein required for VLDL production and secretion from the liver [14]. Prior studies have shown that siRNA-based SVIP silencing in hepatocytes significantly reduces secretion of both [^3^H]-TAG and ApoB100 from SVIP-deficient cells compared to WT cells, thereby demonstrating that silencing of SVIP reduces VLDL secretion from hepatocytes [11]. To further assess how long-term complete loss of the SVIP protein in cells affects VLDL trafficking in depth, we compared the intracellular levels of ApoB100 protein in the SVIP KO and WT cells. The levels of ApoB100 were observed to be significantly higher in the KO cell lysates compared to the WT cells (Figure 2A,B). Even the levels of ApoB48 appeared to be elevated in the SVIP KO cells. Furthermore, a confocal microscopy assay using a BODIPY 493/503 stain that specifically labels intracellular neutral lipids demonstrated an increased FITC signal for BODIPY 493/503 with an increased number of green foci in KO cells (Figure 2C,D). This signals a significantly higher lipid accumulation associated with the loss of SVIP in the KO cells. Additionally, we monitored the levels of [^3^H]-TAG secretion from these cells as a means of observing VLDL secretion. As shown in Figure 2E, at the later time points of 6 h and 24 h, there was a substantial reduction in [^3^H]-TAG secretion from KO cells compared to WT cells (Figure 2E). The level of [^3^H] dpm in the cell culture was measured using a liquid scintillation counter to determine the associated [^3^H]-TAG secretion. Taken together, these data indicate that the loss of SVIP results in KO cells exhibiting a higher intracellular lipid content in comparison to WT cells.

### 2.3. SVIP KO Cells Show a Differential Expression of Genes Involved in Fatty Acid Metabolism and the PPARα and Nrf2 Signaling Pathways

**Figure 3:**
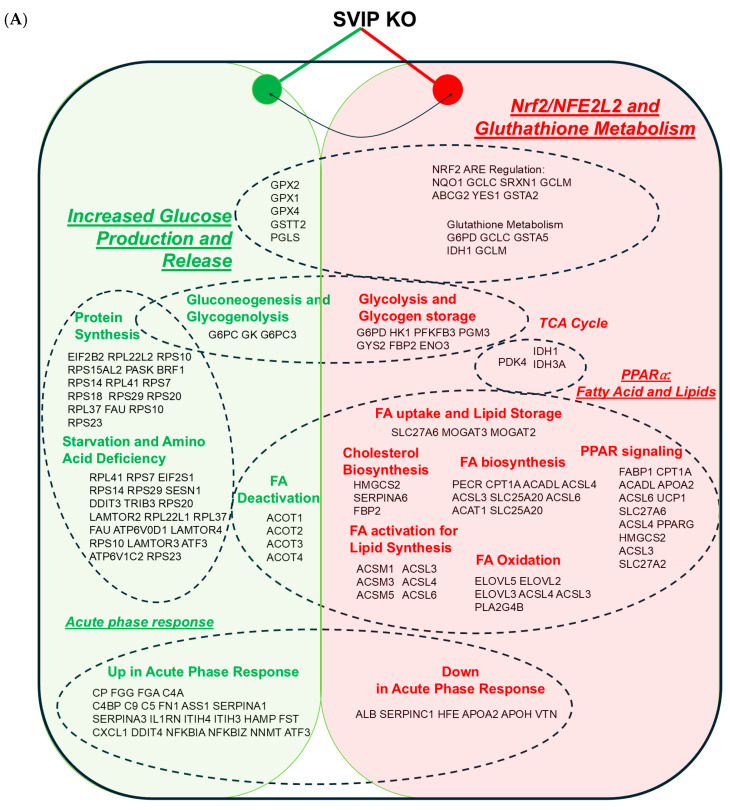

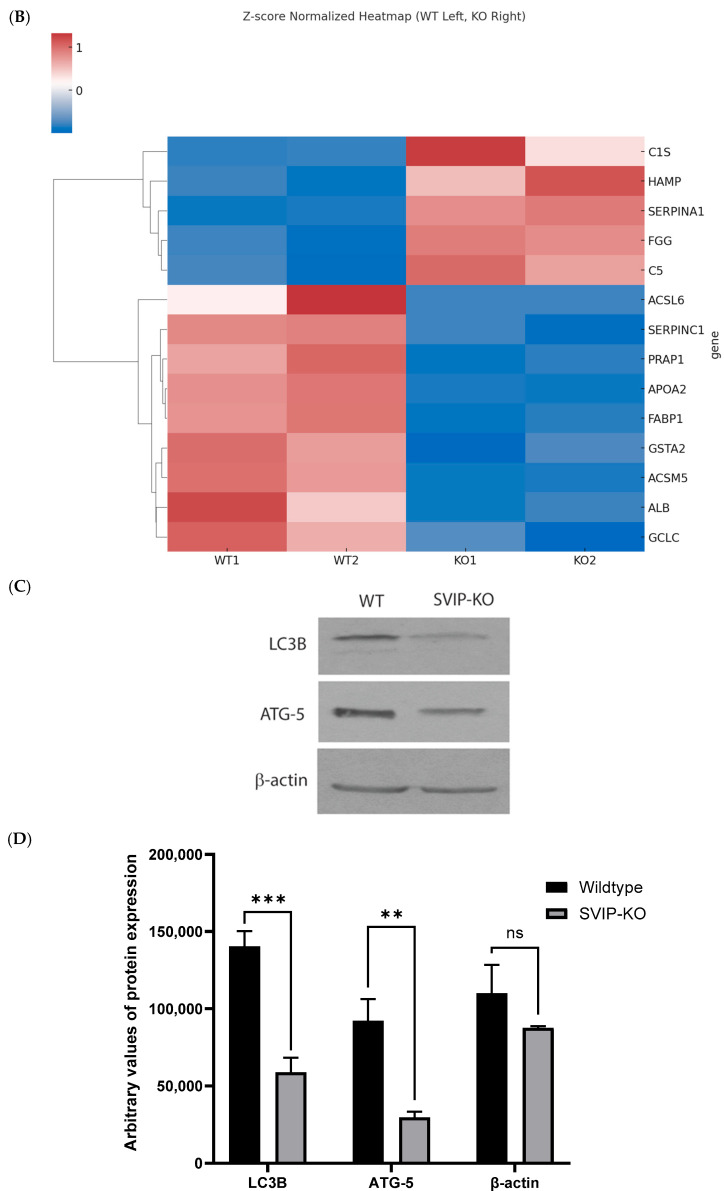
Summary of transcriptional changes in response to SVIP KO hepatocellular carcinoma cells. (**A**) Upregulated genes are represented in green and downregulated genes in red. Major shifts affecting fatty acid synthesis and other pathways are highlighted by dashed-line ovals. For example, loss of SVIP expression results in a shift from FA uptake, activation, synthesis, and oxidation, suggesting impaired PPARα signaling. Simultaneously, the cells favor the activation of glucose (impaired glycogen storage and increased glucose release). The SVIP KO cells show a transcriptional profile reminiscent of acute phase response (APR) of hepatocytes, with a set of genes characteristic for APR being upregulated, while genes that are downregulated during APR are also downregulated in SVIP KO cells. In line with an APR-like response, the cells have increased mRNA expression of genes associated with protein synthesis. These mRNA expression changes indicative of metabolic reprograming include the suppression of Nrf2 target genes and glutathione metabolism. (**B**) Gene expression analysis and heatmap visualization. Differential expression analysis was performed on gene expression data from knockout (KO1, KO2) and wildtype control (WT1, WT2) samples. For each gene, the log_2_ fold change (log_2_FC) was calculated as: log_2_(KO mean WT mean) log_2_(WT mean KO mean). Genes with an absolute log_2_FC greater than log_2_(1.5) ≈ 0.585 were considered biologically relevant. Statistical significance was assessed using a two-sample *t*-test, followed by the Benjamini–Hochberg correction to control the false discovery rate (FDR), with a threshold of FDR < 0.05. For visualization, a heatmap of z-score-normalized expression values was generated. Genes included in the heatmap fell into three categories: (1) custom list of 14 user-specified genes; (2) the top 10 differentially expressed genes, selected based on lowest FDR among those with |log_2_FC| > 0.585; and (3) additional genes meeting the following criteria: FDR < 0.01, |log_2_FC| > 2 (corresponding to ≥4-fold change). Mean expression > 10. Sample labels were reordered to display controls (WT1, WT2) on the left and KOs (KO1, KO2) on the right. Gene rows were hierarchically clustered to highlight co-regulated expression patterns. (**C**) Western blot image confirming a significant reduction in the LC3B and ATG-5 protein levels in the SVIP KO cells compared to wildtype (WT) cells. β-actin was used as a loading control. (**D**) Relative expression levels of the LC3B, ATG-5, and the β-actin proteins in the wildtype and SVIP KO cells. The data are representative of mean ± SD of two independent experiments. (*** *p* = 0.001, ** *p* = 0.004 using 2-way ANOVA, ns = not significant).

To elucidate the factors responsible for an increased intracellular retention of lipids in the SVIP KO cells, we next performed an RNA sequencing study to compare the gene expression profile of the SVIP KO and the WT hepatoma cells. Duplicate samples of each cell line (WT and KO) were used for the RNA sequencing study. The major finding that emerged from the study was the negative impact noted on FA metabolism, especially the mitochondrial long-chain FA β oxidation and the omega-9 FA synthesis in the SVIP KO cells. Interestingly, this downregulation of FA-metabolism-related genes coincides with the partial repression of the PPARα and the Nrf2 signaling pathways. These PPARα and Nrf2 target genes are on an average 2-fold reduced in the SVIP KO cells. PPARα is a nuclear receptor protein functioning as a transcription factor expressed in the liver, among other tissues. It regulates the expression of numerous target genes vital for maintaining cellular lipid metabolism, ranging from mitochondrial, peroxisomal fatty acid oxidation (FAO) to fatty acid uptake and binding, lipolysis, lipogenesis, and glycerol metabolism [15]. Among the highly downregulated genes in the KO cells, inhibition of PPARα signaling manifests in a reduction in important target genes such as LFABP, ACADL, APOA2, SLC27A6, ACSL4, ACSL3, and SLC27A2 (Figure 3A,B). Specifically, genes common to both the fatty acid metabolic pathways and the PPARα signaling pathway, such as ACSM5, ACSL6, PRAP1 and APOA2, were confirmed to be downregulated using real-time quantitative PCR (RT-qPCR) assays (Supplementary Figure S1). Acyl-CoA synthetase medium-chain family member 5 (ACSM5) has been reported to be involved in the first step of fatty acid metabolism via FA ligase and fatty acyl-CoA synthase activity [16]. Downregulation of ACSM5 has been linked to intracellular lipid accumulation [17]. Acyl-CoA synthetase long-chain family member 6 (ACSL6) is an enzyme catalyzing the conversion of fatty acids into acyl-CoA, which is then utilized for lipid biosynthesis and degradation [18]. Similarly, Proline-rich acidic protein 1 (PRAP-1) is a novel lipid-binding protein that promotes lipid absorption by facilitating MTTP-mediated lipid transport [19]. Finally, APOA2, the second most abundant protein of HDL particles, has been shown to be regulated by PPARα signaling, as treatment with PPARα agonists like fibrates increases its expression. Interestingly, PPARα-deficient mice have been reported to demonstrate impaired FA oxidation, lipid accumulation in liver, and hypoglycemia under fasting conditions [20]. Thus, these observations implicate inhibition of PPARα signaling in the SVIP KO cells as a contributing factor to increased intracellular hepatic lipid accumulation.

Moreover, the loss of SVIP also triggers repression of the Nrf2 signaling pathway, which is evident from the reduced expression of key pathway markers such as GSTA2, NQO1, TXNRD1, and G6PD and important glutathione synthesis markers such as GCLC and GCLM (Table 1(1)). Nrf2 is a transcription factor that regulates cellular defense against oxidative and toxic stress, thereby maintaining cellular redox homeostasis [21]. Nrf2 deficiency has been reported to aggravate FA-induced lipid accumulation in hepatocytes both in vivo and in vitro. Its deficiency has also been linked to attenuated autophagy, resulting in reduced lipolysis and increased hepatic lipid accumulation [22]. Studies have reported that the expression of SVIP closely regulates the level of autophagy, especially during the development of CCl4-induced hepatic fibrosis [23]. A cross talk between Nrf2 and SVIP appears to play a role in this process. Notably, SVIP silencing results in the inhibition of autophagy [23]. Interestingly, consistent with the previous findings, both LC3B and ATG-5, the two markers of autophagy, were observed to be significantly downregulated in the SVIP KO cells compared to wildtype cells (Figure 3C,D). This observation supports the possibility of reduced lipolysis in the SVIP KO cells. Thus, the increased intracellular lipid accumulation observed in the SVIP KO cells can be additionally explained by the downregulation of the Nrf2 pathway and the absence of SVIP and its effect on autophagy (Figure 3A,B).

In contrast, the genes overexpressed in the SVIP KO cells show hallmarks of an inflammatory and/or stress response (Table 1(2)). At the onset, it is imperative to keep in mind that the cells are a hepatoma cell line, wherein the cells have already adapted to various stresses. However, in the SVIP KO cells, we observed elevated levels of inflammation markers such as LCN2, FGA, and FGG, the inflammatory cytokine CXCL10, and the stress response gene DDIT4. Overexpression of C4BPB and the anti-inflammatory response protein SERPINA1 likely signals an active immune response. Overall, this gene expression profile mimics the acute phase response (APR) in liver cells, in which the liver cells secrete HAMP, FGG, FGA, SERPINA1, CP, and complement factors such as C1S, C5, and C9, all of which are incidentally upregulated in the SVIP KO cells [24]. On the contrary, expression of genes such as ALB, SERPINC1, and SERPINA6, which are reduced in APR, are also decreased in the KO cells [25] (Figure 3A,B). Interestingly, PPARα has been demonstrated to negatively regulate both pro-inflammatory responses and APR, especially in the rodent models of NASH and atherosclerosis [26,27]. Thus, a muted PPARα signaling likely contributes the APR observed in the KO cells (Supplementary Figure S2).

SVIP has been associated with regulating normal ER function and as an inhibitor of the ER response to unfolded protein stress [13]. Since SVIP is inactivated permanently, the mRNA expression changes may represent an adaptation to chronic ER stress which may manifest in the paradox downregulation of ER stress chaperones such as HSP5A (BiP) and HSP90B1 (GRP94) as well as the upregulation of DDIT3 (CHOP) [28]. The adaptation to a stress state may also explain the elevated levels of ribosomal protein mRNAs in response to SVIP loss. This may help with cellular repair arising from the mimic of the acute phase response we have observed.

The data discussed in this publication have been deposited in NCBI’s Gene Expression Omnibus and are accessible through GEO Series accession number GSE 289707 (https://www.ncbi.nlm.nih.gov/geo/query/acc.cgi?acc=GSE289707) [29].

### 2.4. Knockout of the SVIP Gene Significantly Reduces the Intracellular Levels of the L-FABP Protein

**Figure 4:**
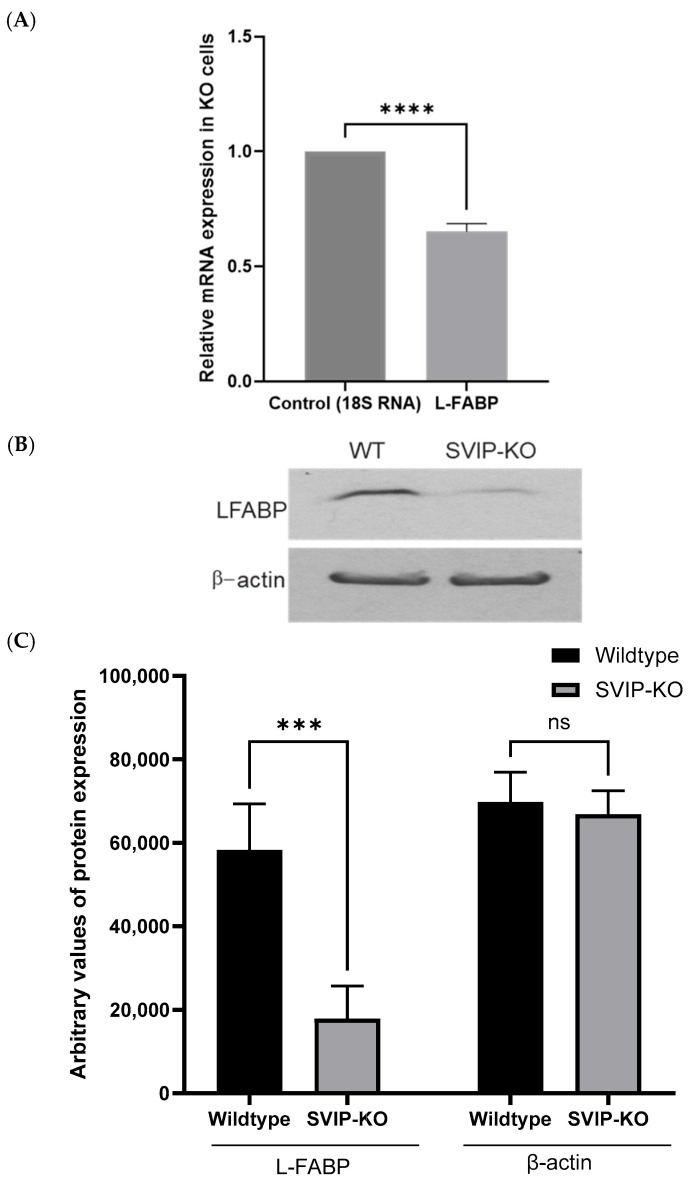
SVIP KO significantly reduces the intracellular levels of L-FABP. (**A**) RT-qPCR assay depicting relative levels of L-FABP mRNA in SVIP KO cells. The data are representative of mean ± SD of three independent experiments. (**** *p* < 0.0001, using unpaired *t*-test). (**B**) Western blot image confirming a significant reduction in the L-FABP protein levels in the SVIP-KO cells compared to wildtype (WT) cells. β-actin was used as a loading control. (**C**) Relative expression levels of the L-FABP and β-actin proteins in the wildtype and SVIP KO cells. The data are representative of mean ± SD of three independent experiments. (*** *p* = 0.0006, using 2-way ANOVA, ns = not significant).

Notably, the RNAseq data additionally revealed a significant downregulation of another important PPARα target gene, L-FABP, in the SVIP KO cells. L-FABP, one of the most abundant cytosolic proteins in both hepatocytes and enterocytes, is critical for fatty acid uptake and is an important regulator of lipid metabolism [30,31]. Because of its ability to bind to a wide range of ligands from long-chain FAs, fatty acyl-CoA, peroxisome proliferators to bile acids, etc., L-FABP is likely involved in a plethora of cellular functions. Importantly, it also protects cells from oxidative stress by binding toxic fatty acids, heme, and cytotoxic molecules. But the interplay of L-FABP and the PPAR signaling pathway by means of L-FABP shuttling its agonists to the nucleus is critical for maintaining hepatic lipid homeostasis. Interestingly both L-FABP and PPARα have been observed to colocalize in the nucleus and demonstrate a direct interaction [32,33]. Furthermore, treatment with PPARα agonists results in increased L-FABP mRNA and protein levels, further confirming it as a bona fide PPARα target [34]. Based on the RNA seq data, we further confirmed that the expression of the L-FABP protein was indeed significantly diminished in the SVIP KO cells using both RT-qPCR and Western blot analysis (Figure 4). As shown in Figure 4A, compared to the wildtype cells, there is about 40% reduction in L-FABP mRNA levels observed in the SVIP KO cells. However, the L-FABP protein is dramatically reduced in the knockout cells, as shown in Figure 4B,C. Thus, the repression of L-FABP protein is plausibly a consequence of a muted PPARα signaling that further contributes to the loss of hepatic lipid homeostasis in the SVIP KO cells.

## 3. Discussion

Hepatic lipid homeostasis and VLDL biosynthesis are strongly interconnected and highly regulated pathways. In addition to the prevalent risk of atherosclerosis, there has also been an increase in hepatic steatosis disorder in patients with dyslipidemia that can eventually progress to cirrhosis [35]. Disturbances in triglyceride utilization via either loss of FA oxidation or secretion of VLDL results in enhanced appearance of lipid droplets in the cytoplasm of hepatocytes [36]. Numerous key players involved in the VLDL assembly and secretion pathways have been identified over the years. Studies from our lab have identified multiple cellular factors including SVIP as an integral part of the VTVs, and these have been shown to play a role in VLDL secretion [11]. To further decipher the role of SVIP in these processes, we have generated a CRISPR-Cas9-mediated SVIP-knockout rat hepatoma cell line. In accordance with previous findings, the SVIP KO cells demonstrated a reduction in VLDL secretion, as determined via the 3H-TAG secretion assay. As a result, we observed that the complete absence of SVIP protein results in enhanced retention of VLDL in the SVIP KO hepatoma cells. This was marked by both an increased expression of ApoB100 protein and neutral lipid staining detected in the knockout cells.

Although our RNAseq dataset consists of two biological replicates per condition, RNA sequencing studies in conjunction with RT-qPCR and Western blotting assays clearly revealed that the SVIP KO cells exhibit muted PPARα and Nrf2 signaling pathways. These pathways are known to regulate genes crucial for maintaining cellular lipid metabolism, mitochondrial and peroxisomal fatty acid oxidation to fatty acid uptake and binding, lipolysis and lipogenesis, and cellular redox homeostasis. Downregulation of these genes has severe repercussions on cellular FA metabolism, inducing intrahepatic lipid accumulation. Notably, PPARα deletion has been reported to promote steatosis and liver inflammation [37]. Our data demonstrating elevated intracellular levels of neutral lipids, which appear as lipid droplets in the SVIP KO cells, mirror the hallmarks of NAFLD. Similarly, lack of Nrf2 signaling has been reported to aggravate FA-induced lipid accumulation, wherein muted Nrf2 has been linked to attenuated autophagy, causing reduced lipolysis and increased hepatic lipid accumulation [22]. Furthermore, we observed that cells lacking SVIP demonstrated a substantial downregulation of another important gene associated with FA metabolism, namely, L-FABP. This is especially significant in the backdrop of muted PPARα signaling, as L-FABP is a PPARα gene target and is also involved in regulating the PPARα signaling pathway due to its ability to bind a large array of ligands. On the contrary, genes overexpressed in the SVIP KO cells show hallmarks of an inflammatory and/or stress response. This data falls in line with studies that have confirmed the role of PPARα in repressing the expression of inflammatory genes and controlling APR [38,39]. Additionally, loss of SVIP also interferes with the ERAD pathway, which can potentially result in ER stress, and thus, the gene expression profile of the SVIP KO cells mimics characteristics of an APR-like state.

In summary, our RNA seq study indicates that the loss of SVIP puts hepatocytes into an APR response like state with increased protein secretion (Secretory Granule Lumen (GO:0034774) significantly up) and a reduced PPARAα and Nrf2 signaling signature. This state is reminiscent to non-alcoholic steatohepatitis (NASH) because of the downregulation of PPARα-related lipid metabolism and inhibition of glycogenesis (GYS2 down), which suggests glucose release or glycolysis (G6P and G6PC3 up, G6PD down). Furthermore, nicotinamide N-methyltransferase (NNMT) was strongly upregulated in SVIP KO cells, reminiscent of what is observed in patients with acute systemic inflammation [40]. Taken together, our findings demonstrate that SVIP is a pivotal cellular protein that plays a crucial role in regulating many significant cellular pathways that have an overarching effect not just on lipid metabolism but also on the overall metabolic state of cells.

## 4. Materials and Methods

### 4.1. Cell Culture

Rat hepatoma cells (McARH-7777) were obtained from American Type Culture Collection (ATCC, Manassas, VA, USA) and cultured in Dulbecco’s modified Eagle’s medium (Corning, Corning, NY, USA) supplemented with 10% FBS (Life Technologies, Waltham, MA, USA) and 1% penicillin–streptomycin (Gibco, Grand Island, NY, USA) in 5% CO_2_.

### 4.2. Antibodies

Primary antibody for SVIP (HPA039807) was obtained from Sigma-Aldrich (St. Louis, MO, USA). Anti-L-FABP antibody (AF1565) was purchased from R&D systems (Minneapolis, MN, USA), and β-actin (8H10D10) antibody was obtained from Cell Signaling Technology (Danvers, MA, USA). Anti-ApoB100 (sc-393636) antibody was purchased from Santa Cruz Biotechnology (Dallas, TX, USA). Anti-mouse, anti-goat, and anti-rabbit secondary antibodies for immunoblotting were purchased from Santa Cruz Biotechnology. Neutral lipid stain BODIPY 493/503 (4,4-Difluoro-1,3,5,7,8-Pentamethyl-4-Bora-3a,4a-Diaza-s-Indacene) was obtained from Thermo Scientific, Waltham, MA, USA.

### 4.3. CRISPR-Cas9 Mediated SVIP Knockout

Two guide RNA sequences (gRNA) targeting the SVIP gene around exon 1, including the promoter region, the CpG island, and parts of intron 1, were designed by considering their high on-target and low off-target efficiency using the http://crispor.tefor.net/crispor.py program (accessed on 25 February 2022). The selected gRNA sequences sgRNA1-CCGCTCCCCTCGCCATTTAG and sgRNA2-ATCAGTGGGGCAACGATGCG were obtained commercially cloned into the CRISPR-Cas9 vector (px458, Addgene) from GenScript. The CRISPR plasmids, each carrying one of the two different gRNA sequences and a green fluorescence protein (GFP) marker gene, were transfected into McARH-7777 rat hepatoma cells using the Lipofectamine 3000 reagent in serum-free media according to the manufacturer’s guidelines. After 4 h at 37 °C and 5% CO_2_, the serum-free medium was replaced with complete media. The cells were then observed under a microscope 48 h post transfection to record the transfection efficiency (via the GFP signal) and to test for any toxicity associated with the reagents. The transfected cells were then GFP-sorted as single cells into 96-well plates. These cells were expanded into small colonies (clonal expansion), and the cells were treated with trypsin and grown in bigger dishes. The colonies obtained were further screened by on-target site PCR following genomic DNA extraction using the following primers pairs: forward: 5′-ACGTACTCCTCGTTTGTGTGG-3′, and reverse: 5′-CGGTAACAGACAGCACCCTT-3′. While many of the expanded clones gave full lengths (1147 bp), few clones demonstrated a significant deletion resulting in a truncated DNA fragment (259 bp). Subsequent DNA sequencing and cDNA sequencing of this fragment confirmed the deletion within the SVIP gene.

### 4.4. Immunoblotting

Cell extracts were prepared in modified radioimmunoprecipitation assay (RIPA) buffer (20 mM HEPES, pH 7.0, 150 mM NaCl, 1 mM EDTA, 1% NP-40, 1% deoxycholate, 0.1% sodium dodecyl sulfate [SDS]) containing complete protease inhibitor cocktail (Roche) and PhosSTOP phosphatase inhibitor cocktail (Roche, Indianapolis, IN, USA). Protein concentrations were determined using a bicinchoninic acid (BCA) protein assay kit (Bio-Rad, Hercules, CA, USA) according to the manufacturer’s instructions. For each sample, 50–60 µg of protein was separated by SDS–polyacrylamide gel electrophoresis (PAGE) and electro-transferred onto nitrocellulose membranes (BioRad). The nitrocellulose membrane was blocked with 10% (*w*/*v*) nonfat dried skimmed milk in PBS-Tween 20. Proteins were detected with different primary antibodies followed by secondary antibodies conjugated with horseradish peroxidase (HRP (Thermo Scientific, Waltham, MA, USA)). Proteins were detected on the membrane with the chemiluminescent reagent SuperSignal West Dura (Thermo Scientific, Waltham, MA, USA). The ImageJ software (v1.52b) was used to perform densitometric analysis of the blots.

### 4.5. Determination of Triglycerides Secretion Using [3H] TAG Secretion Assay

Rat hepatoma wildtype and SVIP KO cells were washed with warm PBS and incubated with [3H] Oleic acid complexed with BSA at a final concentration of 0.4 mM oleic acid for 1 h at 37C. [3H] Oleic acid (Ci/mM) was purchased from PerkinElmer Life Sciences, Waltham, MA, USA. BSA–oleic acid complex was purchased from Sigma, St. Louis, MO, USA. Post-incubation, the radioactive media was replaced with fresh media without oleic acid-BSA complex. A total of 200 µL media was collected in triplicates at different time points, and the radioactivity associated with [3H] TAG was measured as dpm counts using a Tri-Carb 2910 TR liquid scintillation analyzer in 100 µL of collected supernatant (PerkinElmer Life Sciences).

### 4.6. RNA Preparation and Quantitative Reverse Transcription–PCR

Wildtype (WT) rat hepatoma cells (McARH-7777) and their CRISPR-Cas9-mediated SVIP-knockout cells (KO) were untreated or treated with 0.4 mM BSA–oleic acid for 1 h and collected 24 h post-treatment. Total RNA was extracted using an RNeasy Mini Kit (Qiagen, Germantown, MD, USA) according to the manufacturer’s protocol. cDNA was prepared from 500 ng of RNA using a QuantiTect Rev. Transcription Kit (Qiagen, Germantown, MD, USA). Quantitative PCR reactions were set up in MicroAmp optical 96-well reaction plates (Applied Biosystems, Waltham, MA, USA). A QuantiTect SYBR Green RT-PCR Kit (Qiagen, Germantown, MD, USA) was used for amplifying 4 µL of the cDNA with custom primers for 18sRNA and Eif3d from Qiagen and different gene primer pairs (Supplementary Table S1) (Integrated DNA technology, IDT, Coralville, IA, USA). Reactions were amplified using the QuantStudio7Flex instrument (Thermo Fisher Scientific, Wilmington, NC, USA). The relative abundance of transcripts of interest for each cell type were compared using the equation 2–(∆∆Ct), and fold expression was determined relative to the wildtype (WT) control.

### 4.7. RNA Sequencing Studies of Rat Hepatoma Cell Lines

The wildtype and SVIP KO cells were seeded in duplicates on a 6-well plate. Cells were either untreated or treated with 0.4 mM BSA–oleic acid in 2.5% FBS containing DMEM media for 1 h at 37 °C. After an hour, the media were replaced with DMEM media containing 5% FBS, and cells were incubated for 24 h. Cells were harvested, and total RNA from each sample was quantified using a NanoDrop ND-1000 instrument (Thermo Fisher Scientific, Wilmington, USA). A total of 1–2 µg of total RNA was used to prepare the sequencing library in the following steps: 1. total RNA was enriched by oligo (dT)magnetic beads (rRNA removed); 2. RNA-seq library preparation using KAPA Stranded RNA-Seq Library Prep Kit (Illumina, Indianapolis, IN, USA), which incorporated dUTP into the second cDNA strand and rendered the RNA-seq library strand-specific. The completed libraries were qualified with an Agilent 2100 Bioanalyzer (Santa Clara, CA, USA) and quantified by the absolute quantification qPCR method. To sequence the libraries on the Illumina NovaSeq 6000 instrument (San Diego, CA, USA), the barcoded libraries were mixed, denatured to single-stranded DNA in NaOH, captured on an Illumina flow cell, amplified in situ, and subsequently sequenced for 150 cycles for both ends on an Illumina NovaSeq 6000 instrument. Image analysis and base calling were performed using Solexa pipeline v1.8 (Off-Line Base Caller software, v1.8). Sequence quality was examined using the FastQC software (v0.11.7). The trimmed reads (trimmed 5′, 3′-adaptor bases using cutadapt (v1.17)) were aligned to a reference genome using the Hisat2 software (v2.1.0). The transcript abundances for each sample were estimated with StringTie (v1.3.3), and the FPKM value for gene and transcript level were calculated with the R package Ballgown (v2.10.0). The differentially expressed genes and transcripts were filtered using the R package Ballgown. The novel genes and transcripts were predicted from the assembled results by comparing to the reference annotation using StringTie and Ballgown, and then CPAT (v1.2.4) was used to assess the coding potential of those sequences. Then, rMATS (v4.0.1) was used to detect alternative splicing events and plots. Principle Component Analysis (PCA) and correlation analysis were based on gene expression level, hierarchical clustering, and gene ontology pathway analysis. Scatter plots and volcano plots were performed with the differentially expressed genes in the R (v3.5.0), Python (v2.7), or shell environments for statistical computing and graphics.

The data discussed in this publication have been deposited in NCBI’s Gene Expression Omnibus and are accessible through GEO Series accession number GSE 289707 (https://www.ncbi.nlm.nih.gov/geo/query/acc.cgi?acc=GSE289707) [29].

### 4.8. Immunofluorescence Assay

Cells were seeded on cover slips in 24-well plates at a concentration of 40,000–50,000 cells in 500 μL of culture media per well. Before fixation, the culture media were removed followed by three 1× PBS washes (5 min). Cells were then fixed with 4% PFA for 15 min at room temperature. Following washes with PBS, the cells were permeabilized with 0.01% Triton X-100 in PBS for 10 min at room temperature and then blocked with 10% goat serum in PBS-T (0.1% Tween-20 in PBS) for 30 min at room temperature. Cells were then stained with 5 µg/mL of Bodipy 493/503 stain for 1 h at room temperature. Following three washes with PBS (5 min), coverslips were mounted onto slides using a drop of mounting media with DAPI (Southern Biotech, Birmingham, AL, USA). Cells were imaged from randomly selected fields with a Leica SP5 confocal microscop, e (Leica Microsystems Inc., Buffalo Grove, IL, USA) using a 63× objective.

### 4.9. Image Analysis

The acquired images were saved as raw tiff files and imported into Miltenyi’s MACS iQ View software v1.2.2 (Miltenyi, Germany). Nuclear and cell outline segmentation was performed using the DAPI channel and a defined cytoplasmic donut size. The fluorescence intensity of the FITC channel of the BODIPY 493/503 was measured per cell, and the data were exported into GraphPad Prism (v10.4.2).

### 4.10. Statistical Analysis

All statistical analyses were performed using an unpaired *t*-test, Student’s *t*-test, or 2-way ANOVA for independent measures using GraphPad Prism (v10.4.2).

## Figures and Tables

**Table 1:** (**1**) Downregulated genes (at least 20 FPKM in control cells; at least 50% downregulated). (**2**) Upregulated genes (at least 20 FPKM in KO cells; at least 2-fold upregulated).

Name	Fold Change	Average KO	Average CTRL	Function
**(1)**				
**ApoA2**	32.49	11.21	364.45	Plasma lipoprotein assembly, PPARA target
**Pdzk1**	22.27	1.90	42.50	Carnitine transport
**Sult2a6**	15.08	6.22	93.82	Sulfotransferase family member involved in sulfation
**Aadac**	11.23	2.87	32.24	Positive regulation of triglyceride catabolic process
**ApoH**	6.83	36.48	249.31	Component of circulating plasma lipoproteins
**Fabp1**	6.77	72.08	488.10	Regulation of lipid metabolism by PPARα, fatty acid transporters
**Serpina6**	6.56	27.82	182.54	Metabolism of lipids involved in glucocorticoid metabolic process
**Cldn2**	4.88	5.18	25.33	Vitamin D receptor pathway, cell–cell adhesion
**Cryl1**	4.44	6.03	26.80	Carbohydrate metabolism, D-glucuronate catabolic process to D-xylulose 5-phosphate
**Prap1**	4.43	16.31	72.27	Triglyceride binding, DNA damage response, endoplasmic reticulum
**Dpp4**	4.35	16.20	70.44	Synthesis, secretion, and inactivation of Glucagon-like Peptide-1 (GLP-1), ferroptosis
**Gys2**	4.34	9.05	39.28	Glycogen synthesis
**Serpind1**	3.83	5.90	22.64	Formation of fibrin clot (clotting cascade), complement and coagulation cascades
**Atp10a**	3.29	8.54	28.07	ATPase-coupled intramembrane lipid transporter activity, phosphatidylcholine flippase activity
**SVIP**	Inf.	0	11.72	Negative regulation of ERAD pathway, positive regulation of autophagy
**(2)**				
**Paics**	Inf.	55.29	0.00	Nucleotide biosynthesis
**S100g**	10.73	68.41	6.38	Vitamin D receptor pathway
**Lcn2**	7.33	65.58	8.95	Iron uptake and transport
**Fuca2**	5.69	20.62	3.63	Plasma fucosidase
**Cp**	4.33	26.62	6.14	Iron uptake and transport, ferroptosis, metal ion SLC transporters, copper ion binding; up in APR
**Wfdc21**	4.16	58.83	14.13	Antibacterial humoral response
**Fst**	4.00	89.02	22.23	Hepatokine (other hepatokines such as Selenop, Shbg, Smoc1, and Clu are also upregulated), pro skeletal muscle growth, antagonism of activin by Follistatin, up in APR
**Hamp**	3.98	328.06	82.44	Iron metabolism, defense response to bacterium, up in APR
**C4bpb**	3.85	65.24	16.97	Regulation of complement cascade, up in APR
**Cyp2c6v1**	3.70	27.35	7.40	Arachidonate metabolic process, xenobiotic catabolic process, long-chain fatty acid omega-1 hydroxylase activity
**Itih4**	3.43	21.32	6.21	Up in APR
**Ifi27l2b**	3.33	438.58	131.81	Innate immune response
**Arhgef2**	3.32	27.31	8.23	Regulation of RhoA and RAC1 activity, “Innate Immune Sensor”
**Adhfe1**	3.27	20.87	6.37	Oxidation of 4-hydroxybutyrate, aerobic respiration and respiratory electron transport, pyruvate metabolism and citric acid (TCA) cycle, tyrosine metabolism, glutamate catabolic process via 2-hydroxyglutarate
**Ass1**	3.24	28.39	8.75	Citrulline–nitric oxide cycle, amino acid and derivative metabolism, urea cycle, arginine and proline metabolism, up in APR

The top 15 differentially expressed genes identified in the RNA sequencing studies that are upregulated (1) and downregulated (2), respectively, in the SVIP KO cells. Differentially expressed gene and transcript analyses were performed with the R package Ballgown (v2.10.0). Fold change (cutoff 1.5), *p* value (≤0.05), and FPKM (≥0.5 mean in one group) were used for filtering differentially expressed genes and transcripts.

## Data Availability

The RNA sequencing data is available in NCBI’s Gene Expression Omnibus (GEO) and is accessible through GEO Series accession number GSE 289707 (https://www.ncbi.nlm.nih.gov/geo/query/acc.cgi?acc=GSE289707). All data are presented in the manuscript, and raw data (such as immunoblots etc.) will be available upon request.

## Supplementary Materials

The following supporting information can be downloaded at: https://www.mdpi.com/article/10.3390/ijms26157465/s1.

## Abbreviations

The following abbreviations are used in this manuscript:

SVIP	Small valosin-containing protein-interacting protein
L-FABP	Liver fatty-acid-binding protein
Nrf2	Nuclear factor erythroid 2-related factor 2
APR	Acute phase response
COPII	Coat complex proteins II
