# Supplementary material for: Loss of SVIP Results in Metabolic Reprograming and Increased Retention of Very-Low-Density Lipoproteins in Hepatocytes
Source: Int J Mol Sci. 2025 Aug 1;26(15):7465. doi: 10.3390/ijms26157465 (PMC12347863; doi:10.3390/ijms26157465)
Supplement: Supplementary file 1 [file ijms-26-07465-s001.zip › ijms-3687362-supplementary.pdf]

Supplementary Materials

Supplementary Table S1. List of primers for the different genes

Gene	Primers
**ACSM5**	Forward-5’- CTGCCGAAGTGGAACACAGA-3’<br>Reverse- 5’- AGACCAGACCTCTCAGCCAT-3’
**PRAP-1**	Forward-5’- CGAAAGGAAGCCCCTGTCAT-3’<br>Reverse- 5’- GTGTGCTGGAATTGCACCTG-3’
**ACSL6**	Forward-5’- TGTGTTTCACGAGCGGTACA-3’<br>Reverse- 5’- GCCCACTGACTCTCTGTGAC-3’
**ApoA2**	Forward-5’- CACTGTTCCTAGGCCGCATT-3’<br>Reverse- 5’- CTGCCTGTCTCCGAACCAAA-3’
**L-FABP1**	Forward-5’- ATTGCCACCATGAACTTCTCC-3’<br>Reverse- 5’- GCCTTGTCTAAATTCTCTTGCT-3’

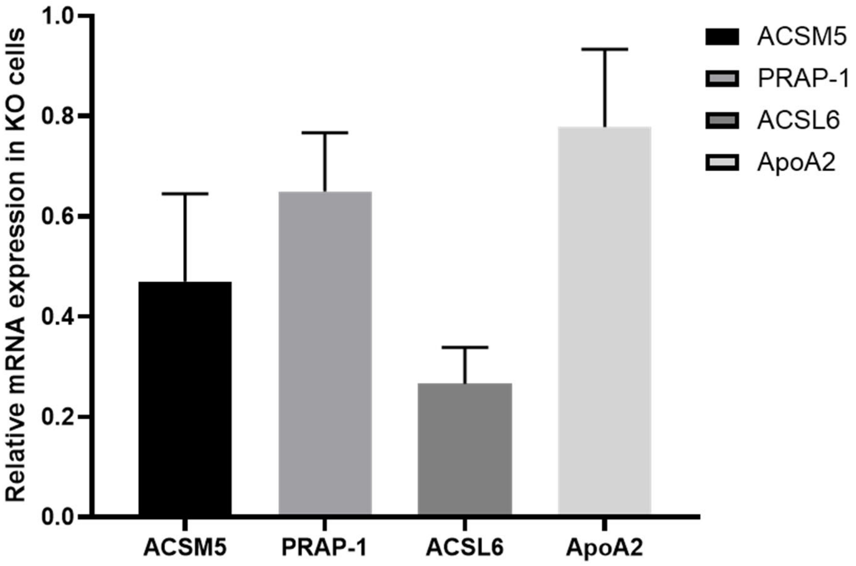

Supplementary Figure S1. RT-qPCR assay depicting relative levels of different PPARα target mRNA in SVIP KO cells. The data are representative of mean ± SD of triplicates of two independent experiments.

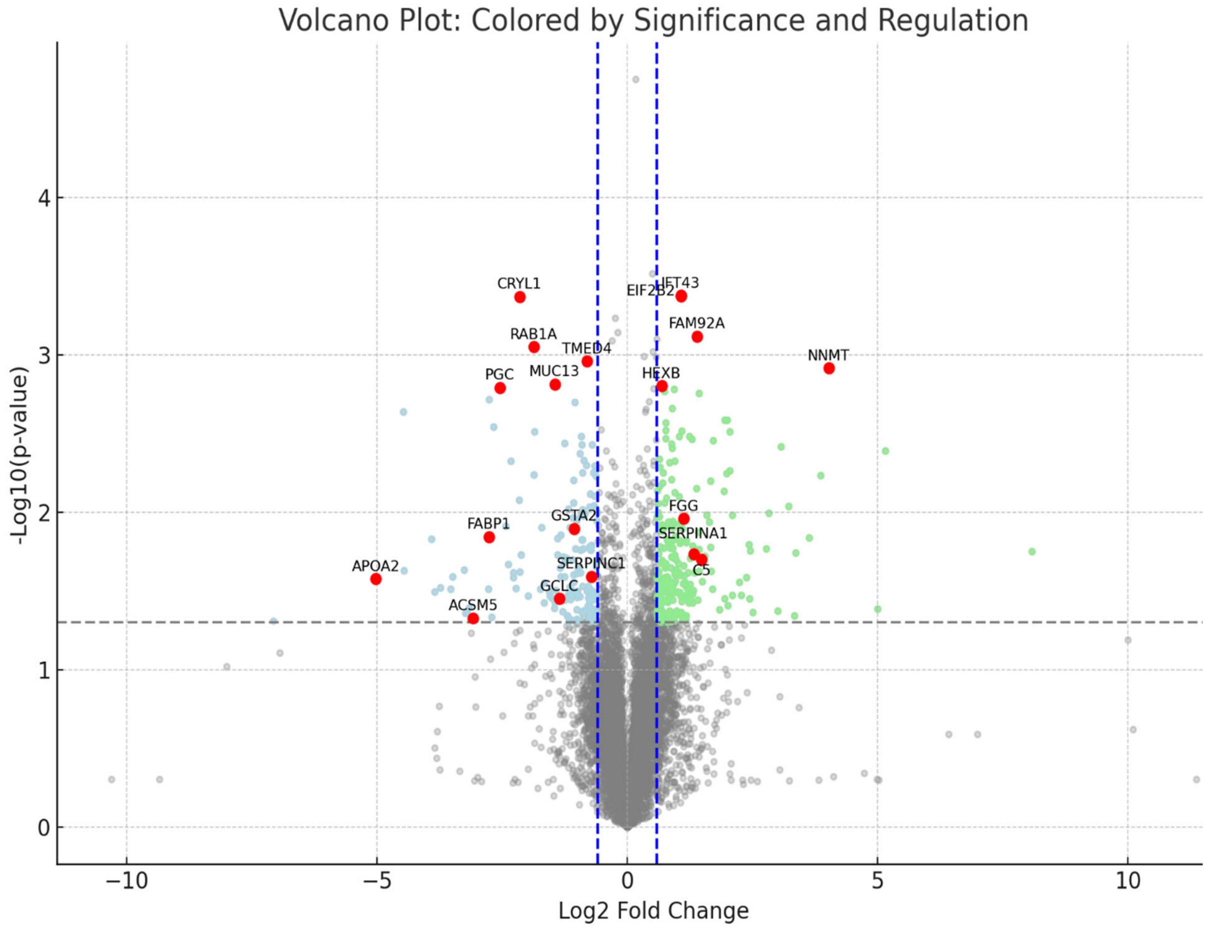

Supplementary Figure S2. Differential Gene Expression Volcano Plot

The volcano plot displays the results of differential expression analysis comparing KO (KO1, KO2) versus control (WT1, WT2) samples. Each point represents a gene, with:

- X-axis: Log_2 fold change (KO vs WT)
- Y-axis: $-Log_{10}$(p-value) from a two-sample t-test
- Gray dots: Non-significant genes
- Light green dots: Significantly upregulated genes (FDR < 0.05, $log_2FC > log_2(1.5)$)
- Light blue dots: Significantly downregulated genes (FDR < 0.05, $log_2FC < -log_2(1.5)$)
- Red dots: Selected genes of interest, either:

1) Provided by the user (custom list), or

2) Among the top 10 most significantly differentially expressed genes

Genes with high fold changes and strong statistical significance are located toward the upper left and upper right corners. Threshold lines indicate the cutoffs for statistical significance (horizontal, $p < 0.05$) and fold change (vertical, $FC > 1.5$). Gene names are labeled for prioritized, red-marked genes.
